# Drugs and Medicines of North America

**Published:** 1884-07

**Authors:** 


DRUGS AND MEDICINES OF NORTH AMERICA.—Volume 1, Number 1. April, 1884—A quarterly magazine devoted to the Historical and Scientific discussion of the Botany, Pharmacy, Chemistry and Therapeutics of the Medical Plants of North America, their constituents, Products and Sophistications. This is a new departure in class literature, and one which will be most valuable. The second number is promised to be of unusual interest. Subscription price $1.00 per annum; J. U. & G. C. Lloyd, 180 Elm street, Cincinnati.

